# Diagnosis of Autism Spectrum Disorder Using Central-Moment Features From Low- and High-Order Dynamic Resting-State Functional Connectivity Networks

**DOI:** 10.3389/fnins.2020.00258

**Published:** 2020-04-28

**Authors:** Feng Zhao, Zhiyuan Chen, Islem Rekik, Seong-Whan Lee, Dinggang Shen

**Affiliations:** ^1^School of Computer Science and Technology, Shandong Technology and Business University, Yantai, China; ^2^Shandong Co-Innovation Center of Future Intelligent Computing, Yantai, China; ^3^BASIRA Lab, CVIP Group, Computing, School of Science and Engineering, University of Dundee, Dundee, United Kingdom; ^4^Department of Brain and Cognitive Engineering, Korea University, Seoul, South Korea; ^5^Department of Radiology and Biomedical Research Imaging Central, University of North Carolina at Chapel Hill, Chapel Hill, NC, United States

**Keywords:** autism spectrum disorder, dynamic functional connectivity networks, resting-state functional MRI, central-moment features, conventional FC network

## Abstract

The sliding-window-based dynamic functional connectivity networks (D-FCNs) derived from resting-state functional magnetic resonance imaging (rs-fMRI) are effective methods for diagnosing various neurological diseases, including autism spectrum disorder (ASD). However, traditional D-FCNs are low-order networks based on pairwise correlation between brain regions, thus overlooking high-level interactions across multiple regions of interest (ROIs). Moreover, D-FCNs suffer from the temporal mismatching issue, i.e., subnetworks in the same temporal window do not have temporal correspondence across different subjects. To address the above problems, we first construct a novel high-order D-FCNs based on the principle of “correlation’s correlation” to further explore the higher level and more complex interaction relationships among multiple ROIs. Furthermore, we propose to use a central-moment method to extract temporal-invariance properties contained in either low- or high-order D-FCNs. Finally, we design and train an ensemble classifier by fusing the features extracted from conventional FCN, low-order D-FCNs, and high-order D-FCNs for the diagnosis of ASD and normal control subjects. Our method achieved the best ASD classification accuracy (83%), and our results revealed the features extracted from different networks fingerprinting the autistic brain at different connectional levels.

## Introduction

Autism spectrum disorder (ASD) is a serious childhood neurodevelopmental disease, characterized by the impairment in social interaction, communication, and many other behavioral and cognitive functions in varying degrees (Geschwind and Levitt, 2007). According to the 2018 community report from the Centers for Disease Control and Prevention (CDCP)^1^, about 1 in 59 American children has been identified with some form of ASD, with about four times more common among boys than among girls. Thus, accurate early diagnosis and timely intervention of ASD, especially for the infants under 12 months old, may have pivotal importance in preventing the progression of detrimental symptoms (Jin et al., 2015). However, ASD is a very complex and highly heterogeneous neurological disorder, which affects many higher-level brain functions and sometimes whole-brain structures, making it challenging for accurate diagnosis. To address this, extensive research efforts (Geschwind and Levitt, 2007; Anagnostou and Taylor, 2011; Jin et al., 2015; Wang et al., 2018) have been dedicated to analyzing the neuroimaging data with different modalities, including structural magnetic resonance imaging (s-MRI) (Wee et al., 2013), functional MRI (fMRI) (Zhao et al., 2018), diffusion tensor imaging (DTI) (Deshpande et al., 2013), and positron emission tomography (PET) (Zürcher et al., 2015), to investigate ASD-related biological or neurological mechanisms. In this way, the respective biomarkers could be identified for characterizing ASD.

Recently, resting-state fMRI (rs-fMRI) uses blood-oxygenation-level-dependent (BOLD) signals to probe brain activity, which has shown great potential in exploring the *in vivo* neuronal underpinnings of ASD (Fornito et al., 2015; Liu et al., 2016; Huang et al., 2018; Zhao et al., 2018). Since BOLD signals are sensitive to the spontaneous and intrinsic neural activities within the brain, re-fMRI can be used as an efficient and noninvasive way for investigating neuropathological substrates of many neurological and psychiatric disorders at a whole-brain system level (Admon et al., 2012; Ganella et al., 2017; Li et al., 2017). Temporal correlation of the BOLD signals between different pairs of brain regions of interest (ROIs) is often used to define brain functional connectivity (FC), which can be used to explore how brain ROIs interact with each other. In practice, FC is often modeled as a FC network (FCN), with each specific brain ROI as a node in the network, and the strength of FC between a pair of brain ROIs as an edge (or link). In terms of both topological structures and connection strength, the differences between normal and disrupted FCN caused by certain pathological attacks reveal potential biomarkers to understand pathological underpinnings of ASD. Therefore, FCN has charted out a promising research direction to investigate the brain’s functional differences between control and disease groups (Zhang et al., 2015, 2016; Qiao et al., 2018).

To date, researchers have developed many FCN models to capture rich information exchange across ROIs so that functional neurological biomarkers can be reliably identified for ASD diagnosis (Jie et al., 2014; Ha et al., 2015a; Plitt et al., 2015). The most commonly adopted FCN, namely, conventional FCN (C-FCN), is usually rooted in the assumption that the strength of FC is temporally stationary in the entire rs-fMRI scan duration (Achard, 2006; Zhao et al., 2018). Under such an assumption, FC is quantified with the correlation (e.g., Pearson’s correlation) between a pair of rs-fMRI time series from two ROIs. As a result, C-FCN captures the functional connectivity between two ROIs in a *static* manner, which unfortunately overlooks the *dynamic* interaction between brain ROIs during the scan period.

In fact, recent studies have demonstrated that the dynamic changes of FC throughout the entire scan time may be an intrinsic property of brain function (Damaraju et al., 2014a; Kudela et al., 2017). Given the increasing evidence that dynamic FC during the entire scan time is very important for understanding the fundamental properties of brain network and the underpinnings of disordered brain connectivity changes, different studies have resorted to dynamic FC networks (D-FCNs) to characterize dynamic changes of FC, as well as the association of these dynamic changes with brain diseases (Damaraju et al., 2014b; Wee et al., 2015; Guo et al., 2017).

**FIGURE 1:**
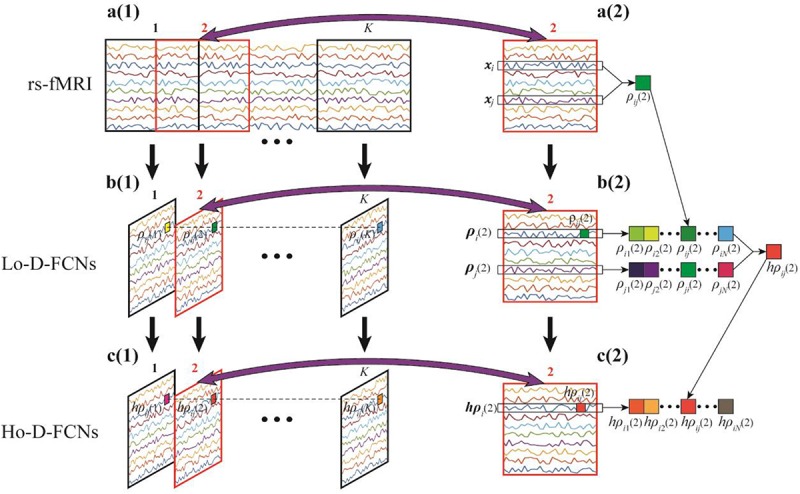
Flow chart of constructing low- (Lo-D-FCNs) and high-order dynamic functional connectivity networks (Ho-D-FCNs), where **(A1)** denotes the resting-state functional MRI (rs-fMRI) time series associated with each region of interest (ROI), **(A2)** denotes the second rs-fMRI subseries based on a sliding window, **(B1)** is the Lo-D-FCNs, **(B2)** is the second subnetwork of Lo-D-FCNs, **(C1)** is the Ho-D-FCNs, and **(C2)** denotes the second subnetwork from Ho-D-FCNs.

The most commonly used strategy of constructing D-FCNs is the sliding-window approach (Hutchison et al., 2013). The detailed contracture process of D-FCNs [i.e., low-order dynamic functional connectivity networks (Lo-D-FCNs), which will be discussed in the following section) is shown in Figure 1. Specifically, the entire rs-fMRI time series from a subject were segmented into multiple overlapping subseries by a sliding window with prefixed window length and step size between two successive windows (Figure 1A1). For each subseries, a FC subnetwork is constructed by calculating the short-term correlation between different ROIs, which is similar to the construction of C-FCN. As an example, the construction process of the second subnetwork is shown in Figures 1A2,B2, where *x*_*i*_ and *x*_*j*_, respectively, denote the average rs-fMRI time series across all voxels within the *i*th and the *j*th ROIs, and their correlation ρ_*ij*_(2) is computed as the FC strength between the *i*th and the *j*th ROIs. In such a way, we can obtain a FC subnetwork (Figure 1B2), which reflects a short-term FC relationship between two ROIs. Repeating the above process, we can obtain a temporal FC subnetwork series, which is called dynamic FC networks (D-FCNs, i.e., Lo-D-FCNs) (Figure 1B1). Obviously, the correlation series (e.g., [ρ_*ij*_(1),ρ_*ij*_(2),⋯,ρ_*ij*_(*K*)] in Figure 1B1) along the scanning time between a pair of ROIs can represent the temporal change of FC between the two ROIs, which indicates that D-FCNs can capture the dynamic properties of FC throughout the scan time and can provide rich discriminative information for ASD diagnosis.

While D-FCNs opens a new avenue for us to comprehensively understand brain activities, it still has the following two issues need to be addressed.

First, D-FCNs cannot reveal the potentially much complex and high-level relationship among multiple ROIs. Similar to C-FCN, D-FCNs is also based on computing pairwise correlation between neural signals, such as Pearson’s correlation and partial correlation, between a pair of rs-fMRIs from two ROIs to estimate the FC strength (Figure 1A2). Although such simple FC network representation has been widely utilized for examining brain functional activity, it dramatically ignores much complex and high-level interactions across multiple ROIs. In such a sense, C-FCN and D-FCNs are referred to as the low-order FCN, and thus, D-FCNs also will be named as Lo-D-FCNs in this paper. Recently, emerging connectomic studies have demonstrated that examining more complex interactions involving multiple ROIs can provide more valuable insights into brain disease fingerprinting and diagnosis (Chen et al., 2016; Zhang et al., 2016, 2017a,b, 2017c; Guo et al., 2017; Morris and Rekik, 2017; Soussia and Rekik, 2018; Zhao et al., 2018). Correspondingly, those FCNs, reflecting complex interactions across multiple ROIs, are referred as the high-order FCN (Ho-FCN).

By far, much attention has been dedicated to construct Ho-FCN models for exploring the interactions among multiple ROIs. For instance, Chen et al. (2016) constructed a Ho-FCN model based on the correlations between each pair of dynamic FC time series from sliding-window-based Lo-D-FCNs. Guo et al. (2017) modeled a Ho-FCN using a minimum spanning tree for Alzheimer’s disease (AD) classification. Based on a more simple and intuitive way, i.e., correlation’s correlation strategy, a new Ho-FCN was developed by Zhang et al. (2016) for more sensitive early AD detection. Different from Lo-FCN or Lo-D-FCNs, Ho-FCN presented by Zhang et al. defines another correlation between two brain regions based on their FC profiles, rather than BOLD signals. Here, the FC profile of a brain region means the traditional low-order FC of this region. In such a way, the correlation’s correlation is able to reveal some interesting information; for example, some brain regions may exhibit stronger correlation with each other in a feature space (defined by FC profile) than the raw neural signal space. Consequently, Ho-FCN is able to provide another source of information for diagnosis (Zhang et al., 2016).

Inspired from the principle of the correlation’s correlation, we construct a novel high-order dynamic FCNs (Ho-D-FCNs) for exploring the high-order dynamic FC relationships among multiple ROIs. Figures 1C1,C2 display the flowchart of constructing Ho-D-FCNs. For each subnetwork from the Lo-D-FCNs, such as the second one shown in Figure 1B2, we regard the correlations series between a ROI and all other ROIs as its short-time FC profile, which reflects the FC relationship between this ROI and all other ROIs in a short scanning time. For example, ρ_*i*_(2) is the short-time FC profile of the *i*th ROI and ρ_*j*_(2) is that of the *j*th ROI (Figure 1B2). Then, the high-order correlation is computed for each pair of ROI based on the associated short-time FC profiles, such as *hp*_*ij*_(2) shown in Figure 1C2. Intuitively, such correlation reflects the relatively shorter time resemblance between a pair of FC profiles from two ROIs (i.e., correlation’s correlation) and thus involves multiple ROIs. By doing so, we can obtain a corresponding high-order subnetwork (e.g., Figure 1C2) from each low-order subnetwork (e.g., Figure 1B2), which reflects how the low-order temporal correlations between different brain ROIs interact with each other during a short scan time. Accordingly, the high-order subnetwork series (Figure 1C1) is referred as Ho-D-FCNs and utilized to reveal some new characteristics for biomarker detection. In fact, the experimental result in *The Most Discriminative Features for ASD Diagnosis* shows that Ho-D-FCNs can provide complementary information to C-FCN and Lo-D-FCNs.

Second, Lo-D-FCNs is sensitive to the chronological order of its subnetworks, which limits its use in comparative studies. Specifically, due to the unconstrained mental activity during the brain resting state, we cannot establish the temporal correspondence among these FC subnetworks from the same temporal window across different subjects. Therefore, the subnetwork series concatenated along scanning time (i.e., Lo-D-FCNs) might be dynamically mismatched across different subjects, which somewhat hinders the investigation and comparison of dynamic FC at a population level. It is noteworthy that Ho-D-FCNs presented in previous section also faces the same problem. By far, no method is proved to be effective in addressing this issue (Zhang et al., 2017a).

Statistical moment methods, including central, Hu, Zernike moments, and so on, have been broadly used in many areas for detecting and deriving various invariant properties of random signals (Hu, 1962; Hung et al., 2006). For the processing of a one-dimensional random sequence generated from a random variable, central-moment method owns the following merits: (Geschwind and Levitt, 2007) although central moment of different order partly characterizes some dynamic properties of a random sequence from its distinct view, their integration can provide a comprehensive characterization of the fluctuation properties of this sequence. (Jin et al., 2015) Most of central-moment features have the clear mathematical interpretability, e.g., for a sequence, its first-order central moment (i.e., mean) can reflect the fluctuation central; second-order central moment (i.e., variance) can reflect the fluctuation level; third-order central moment can reflect the skewness; and the fourth-order central moment can reflect the kurtosis. In theory, the change characteristics of a random sequence can be better represented by central-moment features. Usually, these central-moment features with the range from first- to seventh order are enough for us to analyze and describe the wave profile distribution of a random variable implicated in the sequence (Anagnostou and Taylor, 2011). More importantly, central-moment features are invariant to the temporal order of a sequence. In other words, as one expressional form of a random variable’s probability distribution, central-moment features of a random sequence are immune to the order of its elements (in a mathematical sense).

**FIGURE 2:**
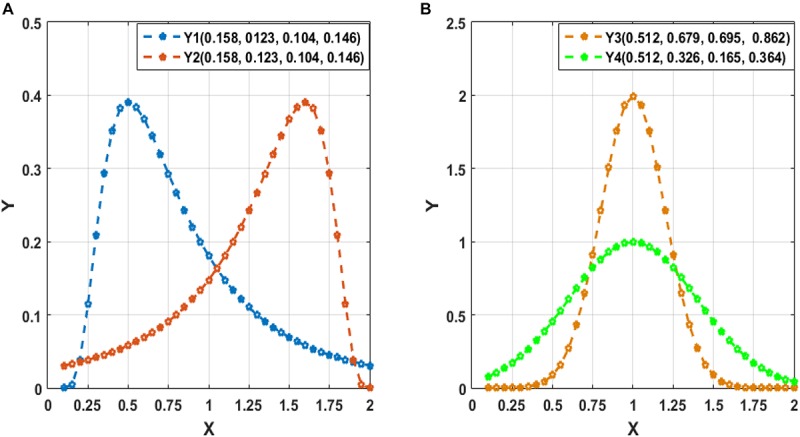
Illustration of the calculated mean, variance, and third- and forth-order central moment (sequentially denoted in the corresponding parentheses) for four sequences Y1–Y4. **(A)** Two sequences (Y1 and Y2) with reversed order. **(B)** Two symmetric sequences (Y3 and Y4) with identical symmetry axis but different fluctuating range.

To clarify the characteristic of central moment, we show the calculated central-moment values of four sequences Y1–Y4 in Figure 2, where the values in the parentheses following each sequence (Y1–Y4) sequentially denote the mean, variance, and third- and forth-order central moment. In Figure 2A, Y1 and Y2 denote two sequences with reversed order. We can see that Y1 and Y2 have the same values of central moment, demonstrating the invariance of central-moment features with respect to the sequence order. In Figure 2B, Y3 and Y4 are two symmetric sequences with identical symmetry axis but rather different fluctuating range. From the calculated central moments for Y3 and Y4, we can see that, except for the mean, the other central moments have noticeable difference, which means that central-moment features are able to reflect the dynamic change of a sequence. Based on the analysis of Figure 2, we can see that the central-moment features is invariant to sequence order and is able to capture the dynamic variation of a sequence.

Inspired by the advantages of central-moment method, we put forward a new approach that employs central-moment technique to excavate the temporal-invariance discriminative features of Lo-D-FCNs. Specifically, we treat each FC correlation time series of a pair of ROIs in a Lo-D-FCNs (such as [ρ_*ij*_(1),ρ_*ij*_(2),⋯,ρ_*ij*_(*K*)] in Figure 1B1), which reflects the temporal changes of FC between two ROIs, as a one-dimensional random sequence that is generated from a random variable, and then, we extract the central-moment features of the sequence for further classification. Similarly, for Ho-D-FCNs, we regard the connection strength (i.e., the connection weight of an edge) series along the scanning time (such as[*h*ρ_*ij*_(1),*h*ρ_*ij*_(2),⋯,*h*ρ_*ij*_(*K*)] in Figure 1C1) as a one-dimensional sequence and extract corresponding central-moment features.

Using the central-moment features, we can summarize the dynamic variation of either low- or high-order FC among multiple ROIs along the scanning time and give a general physiological interpretation to some extent. For example, if the value of the first-order central moment (i.e., mean value) from the FC correlation time series between a pair of ROIs in Lo-D-FCNs or among multiple ROIs in Ho-D-FCNs is relatively large, these ROIs may have strong functional correlation with each other. Similarly, if the value of the second-order central moment (i.e., variance value) is relatively large, it means that the correlations among the corresponding ROIs is very unstable during the whole scanning time; in other words, the periods of high correlation among all the corresponding ROIs may alternate with the periods of low correlation. Contrarily, such an interpretation is very hard to be obtained by directly analyzing Lo-D-FCNs or Ho-D-FCNs due to the large-scale and dynamic network structure.

In summary, there are three parts of contribution in this paper: (Geschwind and Levitt, 2007) proposing new Ho-D-FCNs (never used in previous ASD diagnosis) to reflect high-level connectivity information across multiple ROIs; (Jin et al., 2015) utilizing a central-moment method to capture FC properties derived from Lo-D-FCNs or Ho-D-FCNs without performing chronological time matching; (Anagnostou and Taylor, 2011) employing three multilevel FCN models (i.e., C-FCN, Lo-D-FCNs, and Ho-D-FCNs) to comprehensively investigate complex and multilevel functional associations among brain ROIs.

## Materials and Preprocessing

### Subjects

The rs-fMRI dataset used in this paper was downloaded from a publicly available Autism Brain Imaging Data Exchange (ABIDE) database (Di Martino et al., 2013). To alleviate data heterogeneity, we only consider the rs-fMRI data acquired from 45 ASD patients and 47 normal controls (NCs) with ages ranging from 7- to 15 years old, scanned at New York University Langone Medical Center. All these considered subjects had no excessive head motion with a displacement of <1.5 mm or an angular rotation of <1.5° in any of three directions. The detailed demographic information of these subjects is summarized in Table 1. As shown in Table 1, there were no significant differences (*p* > 0.05) in gender, age, and FIQ between two groups. ASD subjects were diagnosed based on the autism criteria in Diagnostic and Statistical Manual of Mental Disorders, 4th Edition, Text Revision (DSM-IV-TR) (American Psychiatric Association, 2000). More details on the data collection, exclusion criteria, and scan parameters can be obtained from the ABIDE website^2^.

**TABLE 1:** Demographic information of the subjects.

	**ASD**	**NC**	***p*-values**
Gender (M/F)	36/9	36/11	0.2135^a^
Age (mean ± SD)	11.1 ± 2.3	11.0 ± 2.3	0.773^b^
FIQ (mean ± SD)	106.8 ± 17.4	113.3 ± 14.1	0.0510^b^
ADI-R (mean ± SD)	32.2 ± 14.3^c^	–	–
ADOS (mean ± SD)	13.7 ± 5.0	–	–

**ASD, autism spectrum disorders; NC, normal control; M, male; F, female; FIQ, Full Intelligence Quotient; ADI-R, Autism Diagnostic Interview-Revised; ADO, autism diagnostic observation schedule. ^*a*^The p value was obtained by χ^2^-test. ^*b*^The p-value was obtained by two-sample two-tailed t-test. ^*c*^Two patients do not have the ADI-R score.**

### Data Acquisition and Preprocessing

All included subjects were scanned using a 3-T Siemens Allegra scanner at the NYU Langone Medical Center. During the 6 min rs-fMRI scan procedure, most subjects were instructed to relax with their eyes and stare at a white fixation cross at the center of the black screen. Their eye statuses were monitored by an eye tracker. The mean framewise displacement (FD) was computed to describe head motion for each individual. The individuals were excluded if their mean FD is >1 mm (Lin et al., 2015; Ray et al., 2015). On the other hand, head motion effect was further corrected with the Friston 24-parameter model in the following process. The main scanning parameters used in this dataset include the flip angle = 90, 33 slices, TR/TE = 2,000/15 ms, 180 volumes, and voxel thickness = 4 mm.

For rs-fMRI data preprocessing, we used the Statistical Parametric Mapping (SPM8) software^3^. Specifically, the first 10 rs-fMRI volumes were removed to ensure magnetization stabilization. Then, all rs-fMRI volumes were normalized to the Montreal Neurological Institute (MNI) space with the resolution of 3 × 3 × 3 mm^3^. Subsequently, ventricle, global signals were regressed out as nuisance signals, while head motion was corrected with the Friston 24-parameter model (i.e., 6 head motion parameters, 6 head motion parameters from the previous time point, and the 12 corresponding squared items) for decreasing head motion effects (Satterthwaite et al., 2013; Yan et al., 2013). Furthermore, the band-pass filtering (0.01–0.08 Hz) and signal detrending were also performed to avoid physiological noise (Cordes et al., 2001), measurement error (Achard et al., 2008), and magnetic field drifts of the scanner (Tomasi and Volkow, 2010). Finally, the brain was parcellated into 116 brain ROIs using the Automated Anatomical Labeling (AAL) atlas (Tzourio-Mazoyer et al., 2002). Next, the average rs-fMRI time series was calculated for each brain ROI and then represented in a data matrix *X* ∈ *R*^170× 116^, where 170 denotes the total number of temporal image volumes and 116 denotes the total number of all brain ROIs.

## Method

In this section, we mainly detail how to construct our Ho-D-FCNs based on the “correlation’s correlation” principle. As mathematical notations, we use uppercase bold letters (e.g., ***G, C***) to denote FC networks or matrices, lowercase bold letters (e.g., ***x***) to denote vectors, and lower case letters (e.g., *i, j*) to denote scalars.

**FIGURE 3:**
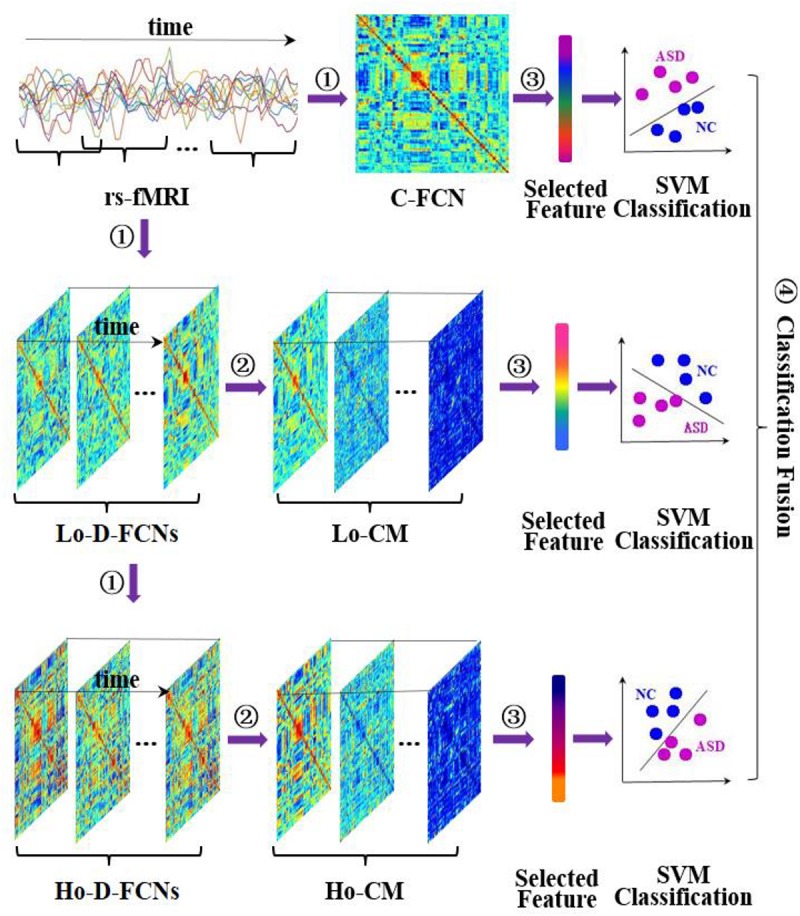
Overview of our proposed classification framework, including four main steps: ① constructing multiple functional connectivity networks (FCNs), ② extracting central-moment features, ③ feature selection, and ④ classification fusion. Lo-CM denotes the central-moment features from low-order dynamic functional connectivity networks (Lo-D-FCNs), and Ho-CM is from high-order dynamic functional connectivity networks (Ho-D-FCNs). The means of other symbols are the same with those presented in *Introduction*.

Figure 3 displays the flowchart of our proposed classification framework, including the following four steps: ① constructing various FC networks, including C-FCN, Lo-D-FCNs, and Ho-D-FCNs; ② extracting the central-moment features, ranging from the first- to the seventh-order, from Lo-D-FCNs and Ho-D-FCNs (central-moment extracted from Lo-D-FCNs and Ho-D-FCNs can be regarded as the network feature since each of its elements is derived from a correlation time series of a pair of ROIs); ③ selecting the most discriminative features in a two-stage feature selection process for reducing feature dimensionality and eliminating irrelevant features to the target classification task; and ④ classification fusion. We construct an ensemble classifier with three linear support vector machines (SVM) classifiers (Cortes and Vapnik, 1995), each being trained with a specific type of FC features. The classification scores by all SVM classifiers are finally fused, by weighted averaging, to predict the target class label (ASD or NC) for a given testing subject.

### Multilevel FC Networks Construction

A network structure can be modeled as a graph comprising a set of vertexes and edges linking them. Let ***G*** denote a FC network where each vertex represents a specific ROI, and each edge is weighted by the strength of FC between its end vertices (i.e., ROIs). Let ***C*** denote the connectivity matrix of ***G***, where each column (resp. row) denotes a specific ROI, and each element of ***C*** denotes the strength of FC between two ROIs. The structure of ***G*** is encoded in ***C***. Next, we will detail how the corresponding connectivity matrices of C-FCN, Lo-D-FCNs, and Ho-D-FCNs are constructed.

#### C-FCN Construction

For each subject, let *x*_*i*_ = (*x*_*i*1_,*x*_*i*2_,⋯,*x*_*iM*_)(*i* = 1,2,⋯,*N*) denote the average rs-fMRI time series across all voxels within the *i*th ROI, where *M* denotes the total number of temporal image volumes, and *N* denotes the total number of all ROIs. We can generate the conventional correlation-based FC network (C-FCN) *G*_*C*_ by a symmetric matrix*C*_*C*_, defined as:

(1) $$C_{C}={\left(\rho_{i\,j}\right)}_{1\leq i,j\leq N},$$

where ρ_*ij*_ denotes the Pearson’s correlation between the average rs-fMRI time series from the *i*th and the *j*th ROIs, defined as:

(2) $$\rho_{i\,j}=\text{corr}\,\left(x_{i},x_{j}\right),$$

It can be seen from Equation (1) that each row or column of *C*_*C*_ denotes the Pearson correlation series between a specific ROI and all other ROIs. Notably, *G*_*C*_ encodes the static interactions between any pair of ROIs during the entire scanning duration, which fails to capture the dynamic nature of neural activity.

#### Lo-D-FCNs Construction

To encode the nonstationary interactions between different ROIs, we adopt the sliding-window strategy to generate Lo-D-FCNs. Specifically, suppose that the length of the sliding window is *T* and the step size between two successive windows is *S*, thus the entire rs-fMRI time series *x*_*i*_ = (*x*_*i*1_,*x*_*i*2_,⋯,*x*_*iM*_)(*i* = 1,2,⋯,*N*) corresponding to the *i*th ROI are partitioned into *K* overlapping segments with a predefined sliding window, where *K* = [(*M*−*T*)/*S*] + 1.

Letting *x*_*i*_(*k*) = [*x*_*i*1_(*k*),*x*_*i*2_(*k*),⋯,*x*_*iT*_(*k*)](*k* = 1,2,⋯,[*cpsbreak*]*K*) denote the *k*th time subseries of*x*_*i*_, we can calculate the *k*th submatric *C*_Lo−D_(*k*) as Equation (1).

(3) $$C_{\text{Lo}-\text{D}}\left(k\right)={\left[\rho_{i\,j}\left(k\right)\right]}_{1\leq i,j\leq N}\;\left(k=1,2,\cdots ,K\right)$$

where ρ_*ij*_(*k*) is computed as:

(4) $$\rho_{i\,j}\,\left(k\right)\,\rho_{i\,j}\,\left(k\right)=\text{corr}\,\left[x_{i}\,\left(k\right),x_{j}\,\left(k\right)\right]$$

Obviously, *C*_Lo−D_(*k*) reflects the interaction between two ROIs during a relatively shorter time period. The submatrix series ${\left\{C_{\text{Lo}-\text{D}}\,\left(k\right)\right\}}_{k=1}^{K}$ along the scanning time describes the temporal change of the connectivity strength for all ROI pairs. The corresponding FCN of ${\left\{C_{\text{Lo}-\text{D}}\,\left(k\right)\right\}}_{k=1}^{K}$ is called Lo-D-FCNs and denoted as*C*_Lo−D_(*k*) (see Figure 3).

#### Ho-D-FCNs Construction

To fully capture high-order functional interactions across brain ROIs, we adopt the “correlation’s correlation” principle (Zhang et al., 2016; Morris and Rekik, 2017; Soussia and Rekik, 2018; Zhao et al., 2018) to generate Ho-D-FCNs. Specifically, for the *i*th ROI of a subject, we can get a correlation series ρ_*i*_(*k*) = [ρ_*i*1_(*k*),ρ_*i*2_(*k*),…,ρ_*iN*_(*k*)] from the *k*th submatrix*C*_Lo−D_(*k*) (see Equation 3). Mathematically, ρ_*i*_(*k*) denotes the *i*th row or column of the symmetric matrix*C*_Lo−D_(*k*). We regard ρ_*i*_(*k*) as the short-time FC profile of the *i*th ROI on the *k*th time subseries, reflecting the correlations between the *i*th ROI and all other ROIs during the *k*th time section. Then, the correlation is computed between the short-time FC profile ρ_*i*_(*k*) of the *i*th ROI and the short-time FC profile ρ_*j*_(*k*) of the *j*th ROI as follows:

(5) $$h\,\rho_{i\,j}\,\left(k\right)=\text{corr}\,\left[\rho_{i}\,\left(k\right),\rho_{j}\,\left(k\right)\right],$$

Obviously, *h*ρ_*ij*_(*k*) denotes the “correlation’s correlation” between the *i*th ROI and the *j*th ROI in the *k*th time section, quantifying how the correlation series ρ_*i*_(*k*) [i.e., the FC profiles ρ_*i*_(*k*) between the *i*th ROI and all other ROIs resemble the correlation series ρ_*i*_(*k*)[i.e., the FC profiles ρ_*j*_(*k*)] between the *j*th ROI and all other ROIs. As a result, *h*ρ_*ij*_(*k*) can reveal more complex relationship between the FC profiles ρ_*i*_(*k*) andρ_*j*_(*k*), not just the original rs-fMRI time series *x*_*i*_(*k*) and*x*_*j*_(*k*). Thus, the correlation coefficient *h*ρ_*ij*_(*k*) can characterize more complex and abstract interactions among multiple ROIs, which occur in a relatively shorter time period. We further define a submatrix *C*_Ho−D_(*k*) in the *k*th time section as follows:

(6) $$C_{\text{Ho}-\text{D}}\,\left(k\right)={\left[\rho \,h_{i\,j}\,\left(k\right)\right]}_{1\leq i,j\leq N},$$

Based on Equation (6), we can construct a Ho-D-FCNs, denoted as*C*_Ho−D_(*k*), where the submatrices series ${\left\{C_{\text{Ho}-\text{D}}\,\left(k\right)\right\}}_{k=1}^{K}$ is regarded as the associated dynamic FC of *C*_Ho−D_(*k*) along the scanning time. Obviously, *C*_Ho−D_(*k*) can capture high-level interactions across multiple ROIs while preserving the dynamic aspect of brain functional activity. Similar to*G*_Lo−D_, Figure 3 displays the main steps for constructing*G*_Ho−D_(*k*).

### Feature Extraction and Selection

With the above-mentioned methods in *Multilevel FC Networks Construction*, three different types of FCN, i.e., *G*_*C*_,*G*_Lo−D_ and *G*_Lo−D_, are obtained to form multilevel representations of functional interactions across multiple ROIs. In this section, we mainly introduce how to extract and select features from these FCNs.

#### Central-Moment Feature Extraction

We note that both FC networks *G*_Lo−D_ and *G*_Ho−D_ are out of temporal synchrony across different subjects. In other words, the *k*th time subseries, $\rho_{i\,j}^{l}\left(k\right)\left(k=1,2,\cdots ,K\right)$ [or$h\,\rho_{i\,j}^{l}\,\left(k\right)$] from the *l*th subject may be inconsistent with $\rho_{i\,j}^{r}\,\left(k\right)$ [or$h\,\rho_{i\,j}^{r}\,\left(k\right)$] from the *r*th subject due to the unconstrained mental activities during resting state. To extract *consistent* dynamic connectomic features across subjects, we propose to extract the central-moment features of *G*_Lo−D_ and carry out the same procedure for*G*_Ho−D_. Specifically, we first construct a FC time series ρ_*ij*_ between the *i*th ROI and the *j*th ROI by concatenating the elements ρ_*ij*_(*k*) (see Equation 3) as follows:

$$\rho_{i\,j}=[\rho_{i\,j}\left(1\right),\rho_{i\,j}\left(2\right),\cdots \rho_{i\,j}\left(k\right),\cdots ,\rho_{i\,j}$$

(7) (K)](1≤i,j≤N, 1≤k≤K),

where ρ_*ij*_ reflects the FC dynamic changes along the scanning time between the *i*th ROI and the *j*th ROI. We calculate its *d*th order central-moment *m*_*ij*_(*d*) of ρ_*ij*_as follows:

(8) $$m_{i\,j}\left(d\right)=\sqrt[{d}]{\frac{\sum_{k=1}^{K}{\left[\rho_{i\,j}\,\left(k\right)-{\bar{\rho }}_{i\,j}\right]}^{d}}{K}}\left(d=1,2,\cdots D\right),$$

where *D* denotes the highest order. We further get a central-moment matrix series ${\left\{M_{\text{Lo}-\text{D}}\,\left(d\right)\right\}}_{d=1}^{D}$ from *G*_Lo−D_ [i.e.,$\,{\left\{C_{\text{Lo}-\text{D}}\,\left(k\right)\right\}}_{k=1}^{K}$ ] by the following definition:

(9) $$M_{\text{Lo}-\text{D}}\left(d\right)={\left[m_{i\,j}\left(d\right)\right]}_{1\leq i,j\leq N}\left(d=1,2,\cdots D\right),$$

It can be seen from Equation (8) that *m*_*ij*_(*d*) is invariant to the element order of ρ_*ij*_ = [ρ_*ij*_(1),ρ_*ij*_(2),⋯ρ_*ij*_(*k*),⋯,ρ_*ij*_(*K*)]. Thus, ${\left\{M_{\text{Lo}-\text{D}}\,\left(d\right)\right\}}_{d=1}^{D}$ is insensitive to temporal asynchrony across subject.

We use the same strategy to derive central-moment matrix series ${\left\{M_{H\,o-D}\,\left(d\right)\right\}}_{d=1}^{D}$ of *G*_Ho−D_ [i.e.,$\,{\left\{C_{\text{Ho}-\text{D}}\,\left(k\right)\right\}}_{k=1}^{K}$ ] using the following formula:

(10) $$M_{\text{Ho}-\text{D}}\left(d\right)={\left[hm_{i\,j}\left(d\right)\right]}_{1\leq i,j\leq N}\left(d=1,2,\cdots D\right),$$

where *hm*_*ij*_(*d*) is computed as follows:

(11) $$hm_{i\,j}\left(d\right)=\sqrt[{d}]{\frac{\sum_{k=1}^{K}{\left[h\,\rho_{i\,j}\,\left(k\right)-{\bar{h\,\rho }}_{i\,j}\right]}^{d}}{K}}\left(d=1,2,\cdots D\right),$$

*h*ρ_*ij*_(*k*) denotes the “correlation’s correlation” between the *i*th ROI and the *j*th ROI in the *k*th time section (see Equation 5). We also give a brief illustration of *M*_Lo−D_(*d*) and *M*_Ho−D_(*d*) construction in Figure 3.

#### Feature Selection Using a Two-Stage Approach

For the *l*th subject, we obtain three types of raw features, i.e., the features $C_{C}^{\left(l\right)}$ of C-FCN, the central-moment features $M_{\text{Lo}-\text{D}}^{\left(l\right)}\,\left(d\right)$ of Lo-D-FCNs, and the central-moment features $M_{\text{Ho}-\text{D}}^{\left(l\right)}\,\left(d\right)$ of Ho-D-FCNs, each of which is a *N*×*N* symmetric matrix. Here, *N* denotes the number of ROIs, and *N* = 116 is set in our case. Since each matrix is symmetric, we only vectorize their lower off-diagonal triangular part to define the feature vector set$\left\{y_{0}^{\left(l\right)},y_{1}^{\left(l\right)},y_{2}^{\left(l\right)}\right\}$, for representing the *l*th subject, where $y_{0}^{\left(l\right)}$, $y_{1}^{\left(l\right)}$, and $y_{2}^{\left(l\right)}$ denote the vectorization of$C_{C}^{\left(l\right)}$, $M_{\text{Lo}-\text{D}}^{\left(l\right)}\,\left(d\right)$, and $M_{\text{Ho}-\text{D}}^{\left(l\right)}\,\left(d\right)$, respectively. The dimensionality of $y_{c}^{\left(l\right)}\left(0\leq c\leq 2\right)$ is$\frac{N\,\left(N-1\right)}{2}$, and it is 6,670 in our case, where *c* denotes the type of feature vector. Obviously, the feature dimensionality is much larger than the total number of subjects. More importantly, many features may be irrelevant to ASD diagnosis.

To remove the redundant features while preserving a small subset of discriminative features that are most likely relevant to ASD pathology, we design a two-stage feature selection strategy. Specifically, in the first stage, for each feature from $y_{c}^{\left(l\right)}\left(0\leq i\leq 2\right)$, we perform a two-sample *t*-test between NC and ASD subjects, due to its simplicity and efficiency. Then, we select the features only with their *p*-values smaller than a certain threshold. In such a way, we can get a preliminary set of features that are highly correlated with the class label, while the rest features not correlated with classification well be eliminated. However, some feature may be still correlated to each other, thus causing feature redundancy. Therefore, to further remove features from these correlated features, we adopt the L_1_-norm regularized least squares regression, known as LASSO (Tibshirani, 1996), to further optimize the feature subset in the second stage. Note that the *t*-test is performed on each feature individually, while LASSO regression considers all features jointly such that the correlation between features can be taken into account. Specifically, let ${\bar{y}}_{c}^{\left(l\right)}\left(0\leq c\leq 2\right)$ denote the features selected by the *t-*test. *I*^(*l*)^ is the class labels of $\,{\bar{y}}_{c}^{\left(l\right)}$, where *I*^(*l*)^ = 1 if the *l*th subject is ASD and *I*^(*l*)^ = −1 if the *l*th subject is NC. Let w_*c*_ represent the weight vector for the feature selection task. Mathematically, the LASSO model can be formalized as energy functional to optimize (Tibshirani, 1996):

(12) $$\text{min}\frac{1}{2}\sum_{l=1}^{L}||I^{\left(l\right)}-<y_{c}^{\left(l\right)},W_{c}>|{|}^{2}+\lambda ||W_{c}|{|}_{1}$$

where ⟨∙,∙⟩ denotes the inner operator, *L* denotes the number of subjects, and λ is a parameter, controlling the model’s sparsity based on the *L*_1_*-*norm regularization. The larger the value of λ, the sparser the model is. In this way, we can jointly achieve sparse feature selection. In other words, those features with nonzero elements of w_*i*_ were eventually retained. Let ${\bar{\bar{y}}}_{c}^{\left(l\right)}\left(0\leq c\leq 2\right)$ denote the final selected set of feature from the original pool of feature vectors$y_{c}^{\left(l\right)}\left(0\leq c\leq 2\right)$.

#### Classifier Learning and Fusing

After selecting the most important features by the two-stage approach, we use SVM with linear kernel for ASD classification. Considering these features ${\bar{\bar{y}}}_{c}^{\left(l\right)}\left(0\leq c\leq 2\right)$ are generated from three FCNs with different level, we train an SVM classifier for each type of features ${\bar{\bar{y}}}_{c}^{\left(l\right)}\left(0\leq c\leq 2\right)$. SVM seeks a maximum margin hyper-plane to separate the samples from two different classes. The empirical risk on the training data and the complexity of the model can be balanced by the hyperparameter γ, thus ensuring good generalization ability on the unseen data. Finally, we can fuse these three SVM classifiers together for making the final result. Specifically, each type of features ${\bar{\bar{y}}}_{c}^{\left(l\right)}$ are used to train a specific classifier. Then, for a test subject, each SVM will output an associated decision score, indicating the probability of that subject belonging to a class. Finally, to obtain classification result, we calculate the weighted average of the three decision scores from these SVM models with weight α tuned for each SVM, which reflects the reliability of corresponding decision score. In Figure 3, we provide a brief illustration of the classifier learning and fusing.

## Experimental Analysis

For evaluating the performance of our proposed method, we adopted a sixfold cross-validation (CV) strategy to perform experiments. For example, all training subjects were randomly partitioned into six subsets (each subset with a roughly equal number of samples), and each time the samples within one subset are selected as the testing dataset, while the remaining samples within the other five subsets are combined together as the training dataset for feature selection and classifier training. For evaluation, we reported the average accuracy of classification results across all six CV cases. Furthermore, to avoid any possible bias in fold selection, the entire sixfold CV process was repeated 10 times, with a different random partitioning of samples each time. Finally, the average statistics of the 10 repetitions was reported. To carry out our proposed method and other competing algorithms, some parameters need to set, such as *p*-values in the two-sample *t*-test model, λ in the LASSO model (*Feature Selection Using a Two-Stage Approach*), and γ and α in the linear SVM model (*Classifier Learning and Fusing*). For fair comparison, we use nested CV to tune the parameters in each method. In particular, for each fold in the above sixfold CV, we perform another fivefold CV on the five subsets, which is used for training for the selection of parameters. The optimal values can be determined by this inner fivefold CV when the average classification accuracy reaches its optimum. Then, the selected parameters are used to learn a model based on the entire training dataset, which is further utilized for classification on the testing dataset. For our approach, we determine the optimal values for the parameters in the following range: *p*–values ∈ [0.01:0.01:0.1],λ ∈ [0.1:0.1:0.7], γ ∈ [2^−5^,2^−4^,⋯,2^5^], and α ∈ [0.1:0.1:0.9].

As usual, we adopt six evaluation measures, i.e., classification accuracy (ACC), sensitivity or true positive rate (TPR), specificity or true negative rate (TNR), positive predictive value (PPV), negative predictive value (NPV), and F1 score, to comprehensively evaluate classification performance. Their definitions are given as follows:

(13) $$\text{ACC}=\frac{T\,P+T\,N}{T\,P+F\,P+T\,N+F\,N},$$

(14) $$\text{TPR}=\frac{T\,P}{T\,P+F\,N},$$

(15) $$\text{TNR}=\frac{T\,N}{F\,P+T\,N},$$

(16) $$\text{PPV}=\frac{T\,P}{T\,P+F\,N},$$

(17) $$\text{NPV}=\frac{T\,N}{F\,N+T\,N},$$

(18) $$\text{F}\,1\,=\frac{2\times T\,P}{2\times T\,P+F\,N+F\,P},$$

where TP, TN, FP, and FN indicate the true positive, true negative, false positive, and false negative, respectively. Note that we treat ASD patients as positive samples and NC as negative samples in this paper.

### The Influence of Parameters on D-FCNs

In the construction of D-FCNs (including Lo-D-FCNs and Ho-D-FCNs) and feature extraction, there are three parameters to tune: (1) sliding window length *T*, (2) the step size between two successive windows *S*, and (3) the order of central moment *d*, which jointly affects the diagnosis accuracy of Lo-D-FCNs and Ho-D-FCNs. To evaluate the impact of these parameters on classification performance and select a suitable combination of parameters for the subsequent multiclassifier fusion, we vary the values of these parameters in specific range (i.e., *T* = [40:10:90],S = [2:2:12],*d* = [1:1:7]) and repeat the classification experiments based on different combinations of these parameters. It is worth noting that when *d* = 1, we use the mean value instead of the first-order moment so that the method can better reflect the sample characteristics.

**FIGURE 4:**
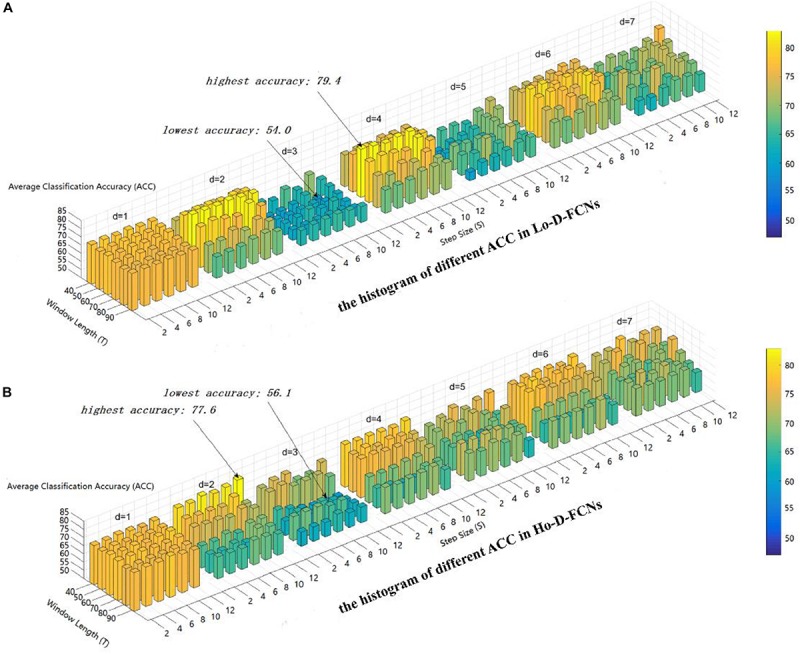
The average classification (ACC) using different combinations of *T*, *S*, and *d* values. **(A)** The histogram of different ACC in Lo-D-FCNs. **(B)** The histogram of different ACC in Ho-D-FCNs.

Here, we use the average classification accuracy (ACC) to evaluate the applicability of parameter combination to ASD diagnosis. Figure 4 displays the ACC achieved by Lo-D-FCNs and Ho-D-FCNs using different combinations of *T*, *S*, and *d* values. The higher the accuracy is, the longer the length and the warmer the color are.

As shown in Figure 4A, the optimal parameter combination for Lo-D-FCNs is *T* = 60,*S* = 2,*andd* = 4, its ACC is 79.4, while the minimum value of ACC is 54.0 when *T* = 60,*S* = 10,*andd* = 3. Likewise, from Figure 4B, we can see that the optimal parameter combination for Ho-D-FCNs is *T* = 40,S = 12,*andd* = 2, its ACC is 77.6, while the minimum is 56.1 when *T* = 70,S = 8,*andd* = d. Therefore, based on Figure 4, we can observe that the classification preformation is rather sensitive to these parameters. For boosting the final classification accuracy, we set these optimal parameters (i.e., *T* = 60,S = 2,*andd* = 4 for Lo-D-FCNs and *T* = 40, *S* = 12, and *d* = 2 for Ho-D-FCNs) as the default parameter for the following experiments.

### Fusion Results of the C-FCN, Lo-D-FCNs, and Ho-D-FCNs

We select the combination of parameters that can lead to the highest ACC from the SVMs of C-FCN, Lo-D-FCNs, and Ho-D-FCNs, respectively, and obtain the final classification result by linear fusion of the SVM ensemble decision scores. In addition to our model, we also added another recently developed high-order FC network approach (Zhou et al., 2018) for comparison. Similar to our approach, this method also used sliding window approach to capture the dynamic variation of FC, and a series of traditional FC networks are constructed. Then, both low-order (termed as LoM) and high-order FC (termed as HiO) networks are constructed by maximum likelihood estimation with the assumption that these D-FCNs follow the matrix variate normal distribution.

Table 2 shows the average classification performance of nine models. Among them, C_C_ denotes the feature derived from the conventional correlation-based FC network (C-FCN), and C_C_ + C_Lo–D_ denotes the fusion of C-FCN and Lo-D-FCNs. The number following C_Lo–D_ denotes the order of central moment used to extract features. For example, C_Lo–D_(1) means the low-order dynamic FC network with mean as central moment. Notice that the constructed LoM network in Zhou et al. (2018) is equivalent to our proposed Lo-D-FCNs when the order of central moment equals to 1, i.e., C_Lo–__D_(1). We also report the standard deviation of the classification accuracy. The best results are highlighted in bold.

**TABLE 2:** Autism spectrum disorder (ASD) classification using different feature types and evaluation measures.

**Model**	**ACC (%)**	**TPR (%)**	**TNR (%)**	**PPV (%)**	**NPV (%)**	**F1 (%)**
C_C_	74 ± 0.04	72 ± 0.23	76 ± 0.01	74 ± 0.05	74 ± 0.08	73 ± 0.07
C_Lo–D_(1)	75 ± 0.12	73 ± 0.14	76 ± 0.29	74 ± 0.23	75 ± 0.08	74 ± 0.12
C_Lo–D_(4)	79 ± 0.15	79 ± 0.10	79 ± 0.53	79 ± 0.38	79 ± 0.07	79 ± 0.12
C_Ho–D_(2)	78 ± 0.06	79 ± 0.49	77 ± 0.24	76 ± 0.09	80 ± 0.25	77 ± 0.11
HiO	72 ± 0.16	71 ± 0.21	73 ± 0.32	72 ± 0.18	73 ± 0.28	72 ± 0.16
C_C_ + C_Lo–D_(4)	80 ± 0.20	78 ± 0.25	82 ± 0.39	80 ± 0.38	79 ± 0.17	79 ± 0.20
C_C_ + C_Ho–D_(2)	78 ± 0.11	79 ± 0.20	77 ± 0.26	77 ± 0.17	79 ± 0.12	77 ± 0.11
C_Lo–D_(4) + C_Ho–D_(2)	81 ± 0.06	82 ± 0.31	80 ± 0.11	80 ± 0.06	83 ± 0.17	81 ± 0.08
C_C_ + C_Lo–D_(4) + C_Ho–D_(2)	**83 ± 0.16**	**82 ± 0.10**	**84 ± 0.46**	**83 ± 0.34**	**83 ± 0.08**	**82 ± 0.13**

*Values highlighted in bold show best results.*

Based on Table 2, we can draw the conclusions below. (1) In terms of ACC and other evaluation measures, the performance of feature types derived from D-FCNs (i.e., Lo-D-FCNs and Ho-D-FCNs) are superior to that of C-FCN, in which ACC is increased by 4 and 5%, respectively, and other performance are also improved accordingly. This result indicates that the sliding-window-based D-FCNs can provide better features for ASD classification. (2) The classification result of ensemble classifier consistently outperforms that of single feature type, which supports the assumption of integrating multiorder connectional features for boosting classification results. (3) The fusion of C-FCN, Lo-D-FCNs, and Ho-D-FCNs achieved the best classification performance, indicating that different-level FCNs can provide complementary relevant information for ASD diagnosis and classification, and the fusion of this information can further improve the classification performance. This result will also be reflected in the following experiments. (4) By comparing our model with the approach proposed in Zhou et al. (2018), we also find that our central-moment-based approach performs better in terms of accuracy. Actually, the performance of HiO is inferior to the corresponding low-order FC network [i.e.,C_Lo–D_(1)], which is consistent with the results given in Zhou et al. (2018). This comparison also verifies the effectiveness of our central-moment features.

### The Most Discriminative Features for ASD Diagnosis

We used *t*-test, followed by LASSO regression, to identify the most discriminative features in C-FCN, Lo-D-FCNs, and Ho-D-FCNs, respectively. In this study, we used the frequency, at which features are selected in all cross-validation cases, to quantify feature relevance to the target classification. The higher the feature frequency, the more reliable and discriminative it is regarded.

**FIGURE 5:**
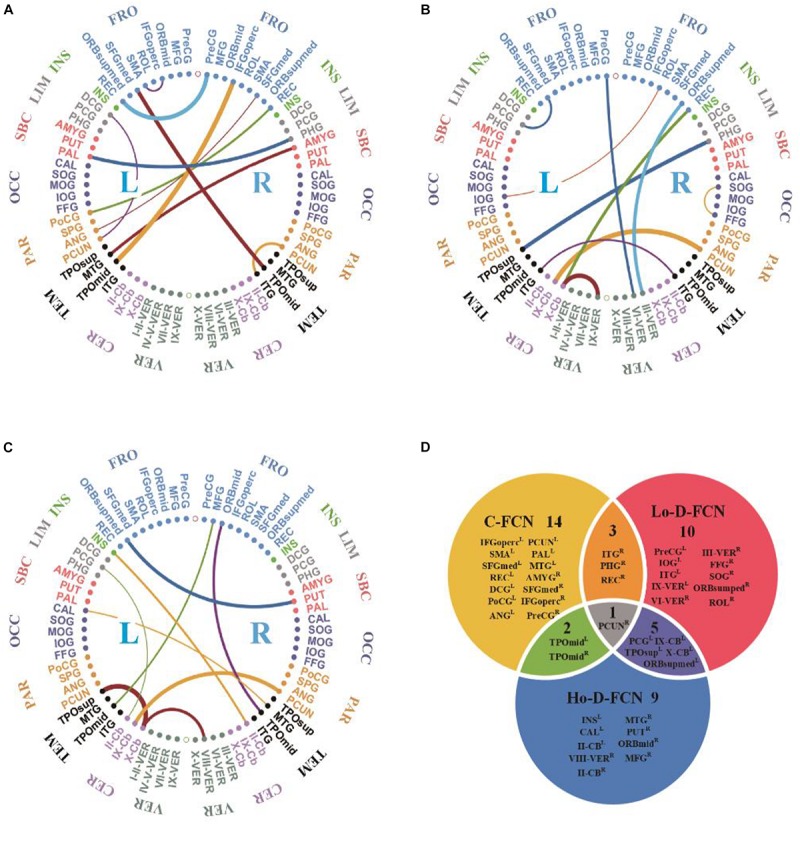
The circular graphs and the involved brain regions of interest (ROIs) of the top 10 discriminative connections selected by our proposed method. **(A)** The correlation-based functional connectivity (FC) network (C-FCN), **(B)** the low-order dynamic FC network (Lo-D-FCNs), **(C)** the high-order dynamic FC network (Ho-D-FCNs), and **(D)** the mutual comparison among three sets of connections. The selection frequency is encoded by the thickness of each connecting curve, i.e., thicker curves indicate higher selection frequency. For brain region abbreviations, please refer to Table 3.

Figures 5A–C visualizes the top 10 most discriminative features of C-FCN, Lo-D-FCNs, and Ho-D-FCNs in the form of circular graphs, where each link corresponds to a connectional feature and represents the correlation between two brain regions (Krzywinski et al., 2009). Figure 5D also shows the mutual comparison among three sets of connections. We use link thickness to encode the degree of their correlation. The thicker the link is, the stronger the correlation is; also, the higher the frequency of the connection selected in cross-validation is, the greater the contribution to the target classification tasks is. For the abbreviations of brain regions in Figure 5, please refer to Table 3. In addition, we mark L (or R) following a brain region (or ROI) name to denote that it lies in the left hemisphere (or the right hemisphere), such as ANG^R^ means the right angular gyrus.

**TABLE 3:** Abbreviations of ROIs selected from conventional functional connectivity network (C-FCN), low-order dynamic FCNs (Lo-D-FCNs), and high-order D-FCNs (Ho-D-FCNs).

**Abbreviation**	**ROI name**	**Abbreviation**	**ROI name**
**FRO: frontal lobe**			
PreCG	Precentral gyrus	MFG	Middle frontal gyrus
ORBmid	Orbitofrontal cortex (middle)	IFGoperc	Inferior frontal gyrus (opercular)
ROL	Rolandic operculum	SMA	Supplementary motor area
SFGmed	Superior frontal gyrus (media)	ORBsupmed	Orbitofrontal cortex (medial)
REC	Rectus gyrus		
**INS: insula**			
INS	Insula		
**LIM: limbic system**			
DCG	Middle cingulate gyrus	PCG	Posterior cingulate gyrus
PHG	Parahippocampal gyrus		
**SBC: subcortical structures**			
AMYG	Amygdala	PUT	Putamen
PAL	Pallidum		
**OCC: occipital lobe**			
CAL	Calcarine cortex	SOG	Superior occipital gyrus
MOG	Middle occipital gyrus	IOG	Inferior occipital gyrus
FFG	Fusiform gyrus		
**PAR: parietal lobe**			
PoCG	Postcentral gyrus	SPG	Superior parietal gyrus
ANG	Angular gyrus	PCUN	Precuneus
**TEM: temporal lobe**			
TPOsup	Temporal pole (superior)	MTG	Middle temporal gyrus
TPOmid	Temporal pole (middle)	ITG	Inferior temporal
**CER: cerebellum**			
II-Cb	Crus II of cerebellar hemisphere	IX-Cb	Lobule IX of cerebellar hemisphere
X-Cb	Lobule X of cerebellar hemisphere		
**VER: vermis**			
I-II-VER	Lobule I, II of vermis	III-VER	Lobule III of vermis
IV-V-VER	Lobule IV, V of vermis	VI-VER	Lobule VI of vermis
VII-VER	Lobule VII of vermis	VIII-VER	Lobule VIII of vermis
IX-VER	Lobule IX of vermis	X-VER	Lobule X of vermis (nodulus)

From Figure 5 and Table 3, we can derive the following. (1) The discriminative connections is not limited to connect the same hemisphere or brain lobe but also includes transhemisphere and all brain lobe, which indicates that the brain function of ASD patients has an abnormal distribution pattern over the whole brain. (2) Most selected brain regions are associated with emotional expression, language understanding, and motion coordination, such as precentral gyrus, middle frontal gyrus, middle cingulate gyrus, posterior cingulate gyrus, amygdala, angular gyrus, and others. These observations are consistent with previous studies (Qiu et al., 2010; Ecker et al., 2015; Ha et al., 2015b; Huang et al., 2018). For example, we found that SFGmed^L^ (Andrews-Hanna et al., 2014), ANG^R^ (Andrews-Hanna et al., 2014), PCUN^L^ (Urbain et al., 2015), CAL^L^ (Perkins et al., 2015), FFG^R^ (Urbain et al., 2016), INS^L^ (Leung et al., 2015; Urbain et al., 2016) contributed more to ASD identification, which is in line with the recent finding reported in the existing literatures. (3) Features selected from C-FCN, Lo-D-FCNs, and Ho-D-FCNs have significant differences, which can be seen from three aspects: first, the selected connected features by each FCN (i.e., the connectional lines in Figures 5A–C are almost entirely different from each other, except for the connected features (IX-Cb^L^-PCUN^R^) selected by both Lo-D-FCNs and Ho-D-FCNs although with different strength; second, according to the affiliation relation of the selected ROIs with respect to corresponding FCNs (Figure 5D), we find that most of the selected ROIs merely belong to one FCN, except one ROI (PUCN^R^) that is jointly selected by all the three FCNs, four ROIs by C-FNC and Lo-D-FCNs (or Ho-D-FCNs), and five ROIs by Lo-D-FCNs and Ho-D-FCNs; and third, the regional distribution of the selected features has huge difference among the three FCNs. For example, the connectional features selected by C-FCN mainly distribute in TEM^L^, PAR^L^, OCC^L^, SBC^L–R^, LIM^L–R^, INS^L–R^, and FOR^L–R^ (Figure 5A). The features selected by Lo-D-FCNs mainly locate in INS^R^, LIM^R^, SBC^R^, OCC^R^, PAR^R^, TEM^R–L^, CRE^L–R^, and VER^L–R^ (Figure 5B) and that of Ho-D-FCNs is in INS^L^, LIM^L^, SBC^L^, TEM^R–L^, and CER^L–R^ (Figure 5C). In summary, the above analysis of difference among three FCNs show that their network infrastructures exist significantly different, which indicate that FCNs of different level can provide complementary information for diagnosis. We think that the main reason causing the huge difference among the three FCNs is that each FCN actually reflects the correlation between brain regions from rather different viewpoints. C-FCN generally captures the static connectional feature since its FC is measured using the whole scanning time rs-fMRI series from any pair of ROIs, while Lo-D-FCNs reveals the dynamically connectional relationship between a pair of ROIs because its FC metric is similar to C-FCN, just using a short-time rs-fMRI series. Compared with C-FCN and Lo-D-FCNs, Ho-D-FCNs uses a vastly different metric to measure the connectional relationship between a pair of ROIs, i.e., using the synchronization of the short-time FC profile between two ROIs to represent their temporary correlation. Therefore, Ho-D-FCNs can reveal some new FC interaction among ROIs, thus providing supplementary information to C-FCN and Lo-D-FCNs.

## Conclusion

In this paper, we proposed new Ho-D-FCNs and used the central-moment method to eliminate the phase mismatch problem of dynamic networks. Through the analysis of feature selection, we believed that the presented Ho-D-FCNs could provide complementary information to our previous research (C-FCN, Lo-D-FCNs). Therefore, we fused these three methods and got the optimal classification results. The experimental results have shown that: (1) Ho-D-FCNs was indeed helpful for mining the relevant information for ASD diagnosis; (2) different level FCNs could provide complementary information and improve the disease recognition rate through fusion; and (3) the central-moment method could help to solve the phase mismatch problem in dynamic networks, including Lo-D-FCNs and Ho-D-FCNs, which were covered in the paper. In addition, in the analysis of feature selection, we also found that most brain regions contributing to classification are related to emotional expression, language understanding, and motion coordination. These findings agree with the behavioral phenotype of ASD (Geschwind and Levitt, 2007; American Psychiatric Association, 2013).

Finally, it should be indicated that the fusion of the three methods based on the decision value of SVM might not adequately integrate the complementary information and thus have an impact on the classification accuracy. Therefore, feature fusion is a direction for future improvement, which will be our future work.

## Data Availability Statement

The datasets generated for this study can be found in the Autism Brain Imaging Data Exchange (ABIDE) database (http://fcon_1000.projects.nitrc.org/indi/abide/abide_I.html).

## Author Contributions

All authors listed have made a substantial, direct and intellectual contribution to the work, and approved it for publication.

## Conflict of Interest

The authors declare that the research was conducted in the absence of any commercial or financial relationships that could be construed as a potential conflict of interest.

## Footnotes

1 https://www.cdc.gov/ncbddd/autism/data.html.

2 http://fcon_1000.projects.nitrc.org/indi/abide/abide_I.html.

3 http://www.fil.ion.ucl.ac.uk/spm/software/spm8/.
